# An Overview of Selenium Uptake, Metabolism, and Toxicity in Plants

**DOI:** 10.3389/fpls.2016.02074

**Published:** 2017-01-11

**Authors:** Meetu Gupta, Shikha Gupta

**Affiliations:** Ecotoxicogenomics Lab, Department of Biotechnology, Jamia Millia IslamiaNew Delhi, India

**Keywords:** selenium, toxicity, sulfate transporters, phytoremediation, biofortification, oxidative stress

## Abstract

Selenium (Se) is an essential micronutrient for humans and animals, but lead to toxicity when taken in excessive amounts. Plants are the main source of dietary Se, but essentiality of Se for plants is still controversial. However, Se at low doses protects the plants from variety of abiotic stresses such as cold, drought, desiccation, and metal stress. In animals, Se acts as an antioxidant and helps in reproduction, immune responses, thyroid hormone metabolism. Selenium is chemically similar to sulfur, hence taken up inside the plants via sulfur transporters present inside root plasma membrane, metabolized via sulfur assimilatory pathway, and volatilized into atmosphere. Selenium induced oxidative stress, distorted protein structure and function, are the main causes of Se toxicity in plants at high doses. Plants can play vital role in overcoming Se deficiency and Se toxicity in different regions of the world, hence, detailed mechanism of Se metabolism inside the plants is necessary for designing effective Se phytoremediation and biofortification strategies.

## Introduction

The breakthrough in selenium (Se) research came into existence in 1957, when Schwartz and Foltz showed that addition of Se in fodder prevented muscular dystrophy and liver cirrhosis in rats (Rayman, 2000). Another turning point in Se research came through the discovery of Se in enzyme Glutathione peroxidase (Rotruck et al., 1973; Behne and Kyriakopoulos, 2001). Since, then the essentiality of Se for animals and human beings came into limelight, and is considered as an essential nutrient in human diet (Hartikainen, 2005). Although, Se performs in variety of functions, its antioxidant and anticancerous properties are of primary concern for mankind (Reid et al., 2008; Wallace et al., 2009; Hatfield et al., 2014). Seleno-aminoacids, selenocysteine (SeCys), and selenomethionine (SeMet) are responsible for most of the reported biomedical effects of dietary Se (Dumont et al., 2006). Selenium acts as the catalytic centre of several selenoproteins, such as glutathione peroxidase (GSHPx), thioredoxin reductase, and iodothyronine-deiodinases hence, it is important in the scavenging of free radicals, protection against oxidative stress, strengthening of immune system etc. (Méplan, 2011; Kaur et al., 2014). Deficiency of Se in human diet causes growth retardation, impaired bone metabolism and abnormalities in thyroid function (Reeves and Hoffman, 2009).

Certain regions of the world are Se-deficient while others are becoming Se-toxic due to natural and anthropogenic activities (Zhu et al., 2009). Both the problems of Se i.e., deficiency and toxicity are harmful to humans and animals (Box 1), hence, all over the world it is called as two edged sword. WHO has recommended 50–55 μg/day Se in human diet all over the world (WHO, 2009; Malagoli et al., 2015; Wu et al., 2015). In humans, Se deficiency occurs when dietary intake of Se is (<40 μg/day) and chronic toxicity is observed above levels of (>400 μg/day) (Winkel et al., 2012). In livestock, the minimal requirement of Se is 0.05–0.10 mg/kg dry forage while, the toxic Se concentration in animal feed is 2–5 mg/kg dry forage (Wu et al., 2015). Keshan and Kashin Beck are severe Se deficiency diseases reported in China and Central Serbia due to low intake of Se in diet at a level of 7–11 μg/day (Renwick et al., 2008; Wu et al., 2015). Toxic symptoms of Se were known before the discovery of this element, when Marco Polo in 13th century observed that in Province of Shanxi, in China, animals died of eating certain Se accumulators (Bodnar et al., 2012). However, toxicity of Se came into limelight after the tragedy of Kesterson wild-life Refuge and Reservoir in San Joaquin Valley in California in 1980, which gave this element a worldwide concern (Winkel et al., 2012). The essentiality of Se to plants is still debatable however; beneficial effect of low doses of Se on plants has been reported by several workers (Cartes et al., 2010; Hasanuzzaman et al., 2011; Saidi et al., 2014).

Box 1 **Role of Se in animals (Mehdi et al., 2013).**

Tragic instance of Se-toxicity in humans was observed in Hubei Province, China after digesting Se rich plants (Fordyce et al., 2000). Livestock is threatened persistently due to weathering of Se-rich bedrocks, and anthropogenic activities like irrigation and mining. Se toxicity lead to a condition called selenosis i.e., garlic odor of the breath, gastrointestinal disorders, hair loss, sloughing of nails, and neurological damage. In extreme selenosis cirrhosis of the liver, pulmonary edema, or even death can occur. Selenium deficiency causes Keshan disease i.e., weakening of heart and also atrophy, degeneration, and necrosis of cartilage tissue in the joints.

About 30 selenoproteins have been identified in animals, which play important roles in antioxidant defense, DNA synthesis, reproduction, immune response, formation of thyroid hormones. Apart from above roles, several studies have reported anticancerous effect of Se against liver, pancreas, prostate, esophagus, and colon cancer. In some studies, cardiovascular risk was found to be associated with low intake of Se and Se-enriched diet found to improve overall health conditions in patients suffering from cardiovascular diseases. Also, Se helps in embryo implantation, placenta retention, reduces infertility by increasing sperm mobility, testosterone, and sperm synthesis. Selenoproteins like Glutathione peroxidase, Thioredoxin reductase play important role as antioxidants in maintaining intracellular redox potential. Deiodinase is involved in thyroid hormone metabolism. Selenoprotein P transports Se between tissues and is an important extracellular antioxidant constitutes about 50% of plasma Se.

Selenium was discovered accidentally by Swedish chemist Jons Jacob Berzelius in 1817. The word selenium is derived from Greek word “selene” which means moon (Reilly, 2006; Bodnar et al., 2012). Selenium is a metalloid belongs to group 16 (Oxygen Family) of the periodic table. Being member of the same group of the periodic table, ionic radius of Se and S are closer, hence, physico-chemical properties of both elements are similar to each other (Bodnar et al., 2012). Due to its semiconductive properties, Se is widely used in making electronic and electrical goods. In nature, it occurs as pyrites of Cu, sulfides of Pb, Au, and Cu. It is also a byproduct of metallurgical operations, and widely used in glass industry, paints, lubricating oil, pigments, food supplements, agricultural products etc. (Bodnar et al., 2012; Mehdi et al., 2013).

**Figure 1:**
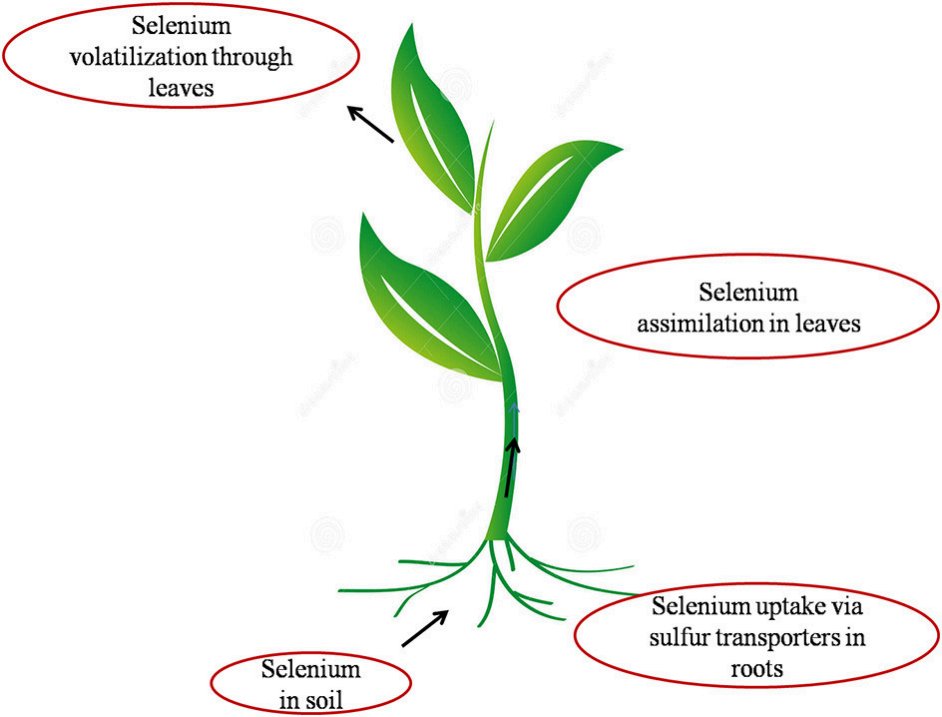
**Pictorial representation of the interface of Se with soil, plant and atmosphere**. Selenium present in soil is transported inside the plant through sulfate transporters present in the plasma membrane of root cells. It is then assimilated to organic Se via sulfur metabolic pathway inside the plant and volatilized as DMSe (Dimethylselenide) and DMDSe (Dimethyldiselenide) into atmosphere.

Plants are the main source of dietary Se for human beings and animals hence, knowledge of the Se compounds in plants is crucial (Dumont et al., 2006). Selenium shares similar chemical properties to sulfur, hence taken up inside the plants via sulfate transporters and assimilated by sulfur assimilating pathway (Sors et al., 2005; Dumont et al., 2006) as shown in (Figure 1).

## Selenium in environment

Selenium occurs naturally in sedimentary rocks formed during the carboniferous to quaternary period (White et al., 2004). Worldwide, average Se concentration in soils is 0.4 mg/Kg however, in seleniferous soils elevated levels of Se (>2–5000 mg/Kg) are found (Hartikainen, 2005). The occurrence of Se in soil depends upon type of soil, organic matter and rainfall (Sors et al., 2005).

**Figure 2:**
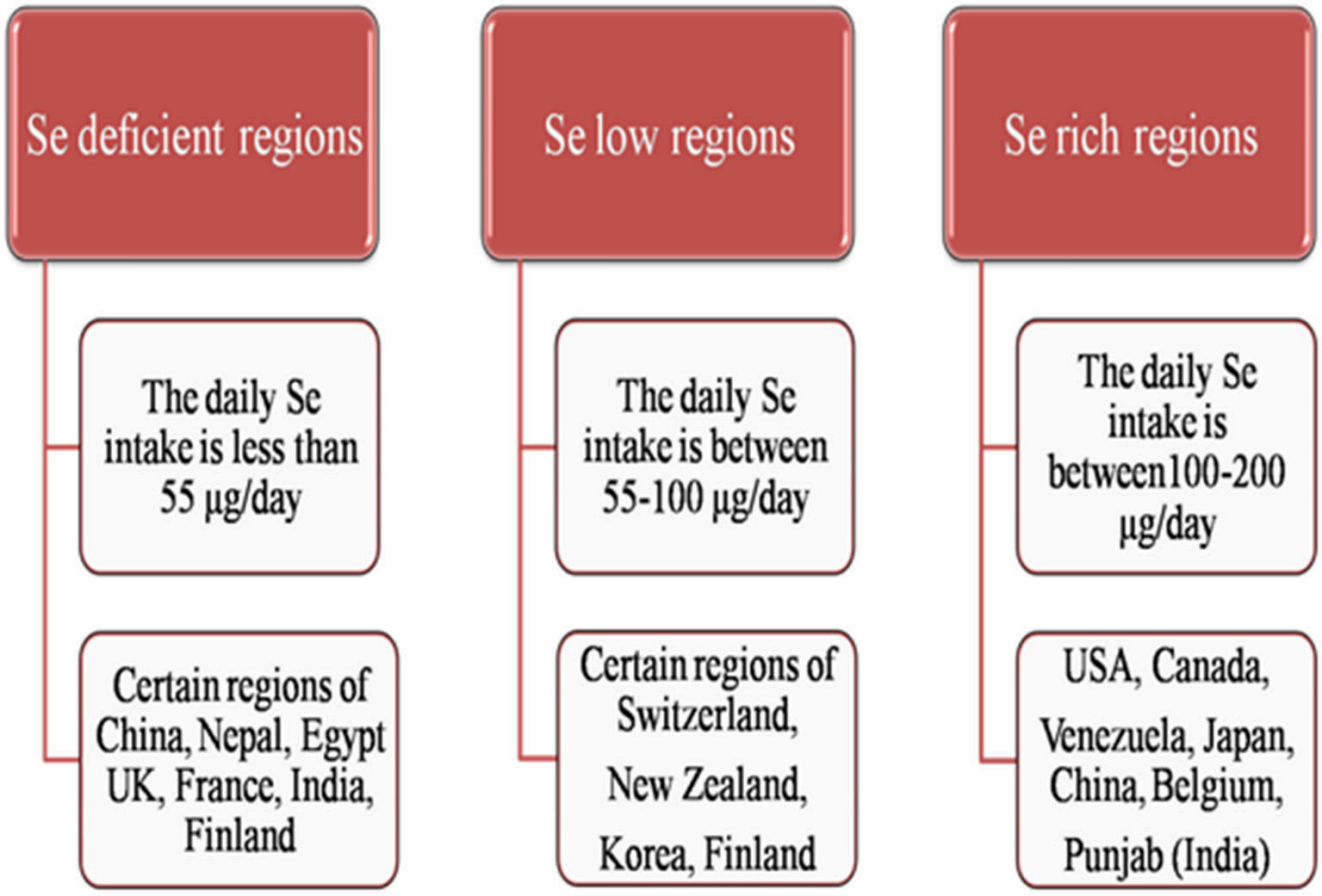
**Outline of occurrence of Se in different regions of world as Se-deficient, Se-low and Se-rich regions**.

Mountainous countries like Finland, Sweden, and Scotland are generally deficient in soil Se content whereas Shale soils and dried regions of the world are Se-rich regions. Countries like UK, France, India, Belgium, Brazil, Serbia, Slovenia, Spain, Portugal, Turkey, Poland, Germany, Denmark, Slovakia, Austria, Ireland, Greece, Netherlands, Italy, China, Nepal, Saudi Arabia, Czech Republic, Croatia, Egypt, Burundi, and New Guinea are reported to have Se deficient areas (Zhu et al., 2009; Yin and Yuan, 2012; Figure 2) while, Se rich regions are North-East region of Punjab in India (Bajaj et al., 2011), Enshi district in Hubei province region in China (Feng C. X. et al., 2009), State of Para in Brazilian Amazon (Lemire et al., 2009), Japan, Greenland (Fordyce et al., 2005), USA, Venezuela and Canada (Yin and Yuan, 2012; Figure 2). About 80% of the world's total Se reserves are located in Peru, China, Chile, the United States, Canada, Zambia, Philippines, Zaire, Australia, and New Guinea (Liu et al., 2011). Although China is ranked the fourth in Se reserves worldwide (after Canada, United States, and Belgium), Se-deficiency occurs in a geographic low-Se belt stretching from Heilongjiang Province in the northeast to Yun-nan Province in the southwest, affecting 71.2% of Chinese land (Zhu et al., 2009). Almost 40 countries in the world have limited natural Se resources. Certain areas in countries like Switzerland, Korea, Australia, New Zealand, and Finland are also identified as Se adequate to Se low regions (Wu et al., 2015; Figure 2).

Selenium level in public water supplies should not increase more than 10 μg/L (NAS, 1976, 1977; Gore et al., 2010). In underground water, Se concentration is increasing due to overuse of Se-containing fertilizers (Winkel et al., 2012) and has reached upto estimated concentration of 0.12 μg/L in Belgium, 2.4–40 μg/L in France (Mehdi et al., 2013), and 341 μg/L in Punjab (Bajaj et al., 2011). In fresh and sea water, its concentration varies from 4000 to 12000 μg/L. WHO has recommended 10 μg/L as the lower intake limit of Se in drinking water (Mehdi et al., 2013). Natural and anthropogenic activities add Se into atmosphere, and play an important role in biogeochemical cycling of Se in environment (Winkel et al., 2012). Natural activities include, forest fire, soil erosion whereas, anthropogenic activities are burning of fossil fuels, tires, papers etc. (Mehdi et al., 2013). In atmosphere, Se is mostly present as volatile organic compounds i.e., DMSe, DMDSe, methaneselenol, and volatile inorganic compound (SeO_2_). SeO_2_ is unstable and converted into selenious acid. The Se content in air is generally low, as compared to soil and water, and ranges between 1 and 10 ng/m^3^ (Mehdi et al., 2013).

Selenium content in food sources varies from plant to plant. It depends upon Se uptake and accumulating capacity of plant, soil Se content, which varies according to geographical locations and presence of other elements in soil (Dumont et al., 2006; Bodnar et al., 2012; Mehdi et al., 2013). Fruits generally contain low amount of Se as compared to vegetables. Selenium content in cereals ranges between 0.01 and 0.55 μg/g, and in milk and dairy products varies between 0.001 and 0.17 μg/g FW (Dumont et al., 2006). Brazil nuts, *Brassica* species, garlic effectively accumulate Se, and are rich sources of Se in diet (Dumont et al., 2006; Bodnar et al., 2012).

## Selenium uptake and accumulation in plants

Selenium exists as inorganic and organic forms in nature. Inorganic forms are selenate (${\text{SeO}}_{4}^{2-}$), selenite (${\text{SeO}}_{3}^{2-}$), selenide (Se^2−^), elemental Se, and the major organic forms are SeCys and SeMet (Sors et al., 2005; Bodnar et al., 2012; Wu et al., 2015).

### Selenium uptake

The uptake, translocation and distribution of Se depends upon plant species, phases of development, form and concentration of Se, physiological conditions (salinity and soil pH) and presence of other substances, activity of membrane transporters, translocation mechanisms of plant (Zhao et al., 2005; Li et al., 2008; Renkema et al., 2012). Selenate (${\text{SeO}}_{4}^{2-}$) is the most prevalent form of bioavailable Se in agricultural soils, and more water soluble than selenite (Sors et al., 2005; Missana et al., 2009). In alkaline soils, Se mostly exists as selenate whereas, in acidic soils it exists as selenite. Both forms of Se differ in terms of their mobility and absorption within the plant and are metabolized to form selenocompounds (Li et al., 2008). Translocation of an ion or molecule to shoot tissue depends on the rate of xylem loading and the rate of transpiration (Renkema et al., 2012). Kikkert and Berkelaar (2013) evaluated mobility of Se species in Canola and Wheat by studying translocation factor and was in the following order: selenate> SeMet> selenite/SeCys. Selenium uptake in plants is mediated by transporters present in root cell membrane. Selenite is found to be transported by phosphate transport mechanism (Li et al., 2008) whereas, selenate through sulfate transporters and channels (Feist and Parker, 2001; Zhang et al., 2003).

Nutritional status inside and outside the plant; determines the preference of these transporters for selenate and sulfate (White et al., 2004). Under high external sulfate concentrations selectivity of these transporters for Se decreases, and inducible sulfate transporters showed higher selectivity for sulfate over selenate than constitutive active sulfate transporters (White et al., 2004). In *Arabidopsis thaliana* sulfate transporters, SULTR1;2 and SULTR1 found to transport selenate inside the plant (El Kassis et al., 2007). In other study enhanced selenate resistance in SULTR1;2 lacking *Arabidopsis* plants but not SULTR1 suggests that SULTR1;2 is the predominant transporter for uptake of selenate into the plant root (Shibagaki et al., 2002; El Kassis et al., 2007). In *Triticum aestivum*, sulfur starvation enhanced Se uptake (Li et al., 2008). According to several workers, selenite uptake is known to be done through passive diffusion (Terry et al., 2000; Ellis and Salt, 2003), however, in another study it is mediated by active transport as uptake of selenite was significantly inhibited by metabolic inhibitor CCCP (Li et al., 2008). Terry et al. (2000) reported non-involvement of membrane transporters in selenite uptake. Li et al. (2008) reported enhanced selenite uptake in phosphorous deficiency, which indicates selenite uptake by phosphate transporters, and supports the earlier findings of decreased selenite uptake under increasing phosphate concentrations.

### Se accumulation in plants

Generally, Se concentration found to be higher in younger leaves as compared to older ones during seedling growth (Cappa et al., 2014; Harris et al., 2014). Inside the plant cells, Se is mostly accumulated in their vacuoles (Ximénez-Embún et al., 2004; Mazej et al., 2008) and can be effluxed through sulfate transporters present in the tonoplast (Gigolashvili and Kopriva, 2014). The Fabaceae constitutes greatest number of Se species known to hyperaccumulate Se.

**Figure 3:**
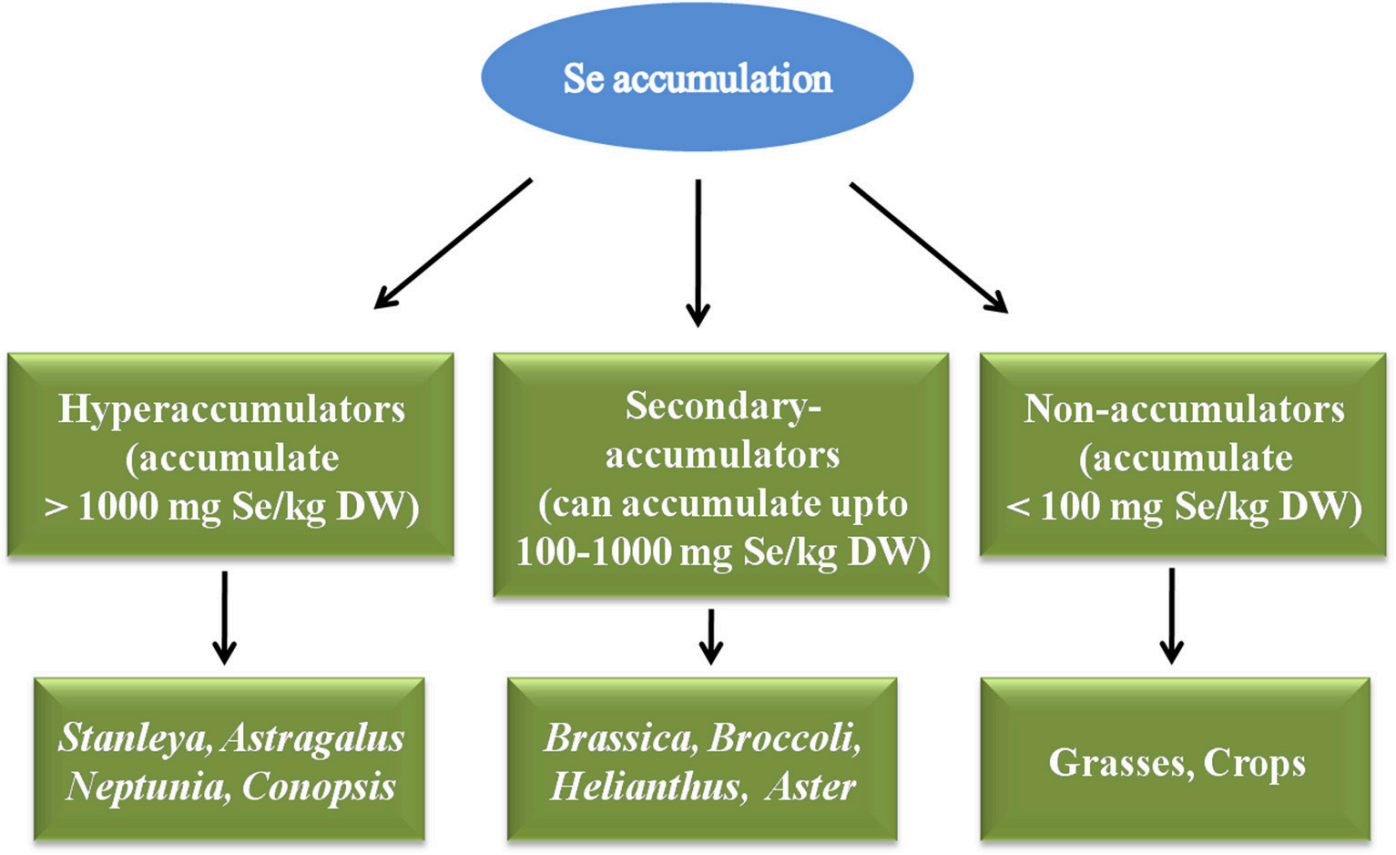
**Classification of plants depending upon Se accumulation as hyperaccumulators, Secondary accumulators and non-accumulators**.

Plants have been classified as hyperaccumulators, secondary-accumulators, and non-accumulators depending upon Se accumulation inside their cells (Galeas et al., 2007; Bodnar et al., 2012; Figure 3). Hyperaccumulators accumulate higher amounts of Se in their cells i.e., >1000 mgSe/Kg DW and thrive well in Se rich regions of the world. They have methylated forms of SeCys and SeMet, which confer Se tolerance of these plants, and can be vaporized further as dimethyldiselenide (DMDSe). Hyperaccumulators include *Stanleya, Astragalus species, Conopsis, Neptunia, Xylorhiza* etc. Secondary-accumulators accumulate Se and show no signs of toxicity upto 100–1000 mgSe/Kg DW, for e.g., *Brassica juncea, Brassica napus*, Broccoli, *Helianthus, Aster, Camelina, Medicago sativa* etc. Non-accumulators are those plants which accumulate less than 100 mgSe/Kg of their DW, and if they grow on Se-rich soils they can't survive, show retarded growth, volatilize Se as dimethylselenide (DMSe) for e.g., grasses and crops (Galeas et al., 2007; Bodnar et al., 2012). When non-accumulators are enriched with Se, it is sequestered quickly in vacuoles of mesophyll cells of leaves (Ximénez-Embún et al., 2004; Mazej et al., 2008). Selenium content in common Se-enriched crops and cereals after fortification with different concentrations of Se have been given in Table 1.

**Table 1:** **Common Se-enriched crops, cereals after fortification with different concentrations of Se**.

**SNo**.	**Crops, vegetables, fruits**	**Se-enriched plant part studied**	**Se used for biofortification (mg/kg or mg/L)**	**Se accumulated (mg/kg)**	**References**
1	Rice	Grain	2.850 mg/kg	1.3–3.3	Sharma et al., 2014
2	Chick Pea	Sprouts	50 mg/L	1.134	Zhang et al., 2012
3	Sorghum	Seeds	75 mg/L	2.1	Djanaguiraman et al., 2010
4	Soyabean	Bean	130 mg/kg	75	Chan et al., 2010
5	Barley	Grain	0.20 mg/kg	0.047	Yan et al., 2011
6	Kale	Sprouts	60 mg/L	155	Thosaikham et al., 2014
7	Broccoli	Sprouts	60 mg/L	467	Thosaikham et al., 2014
8	Pear	Fruit	1 mg/L	0.199	Pezzarossa et al., 2012
9	Lettuce	Shoots	below 2.8 mg/L	43	Hawrylak-Nowaka, 2013

## Selenium metabolism in plants

**Figure 4:**
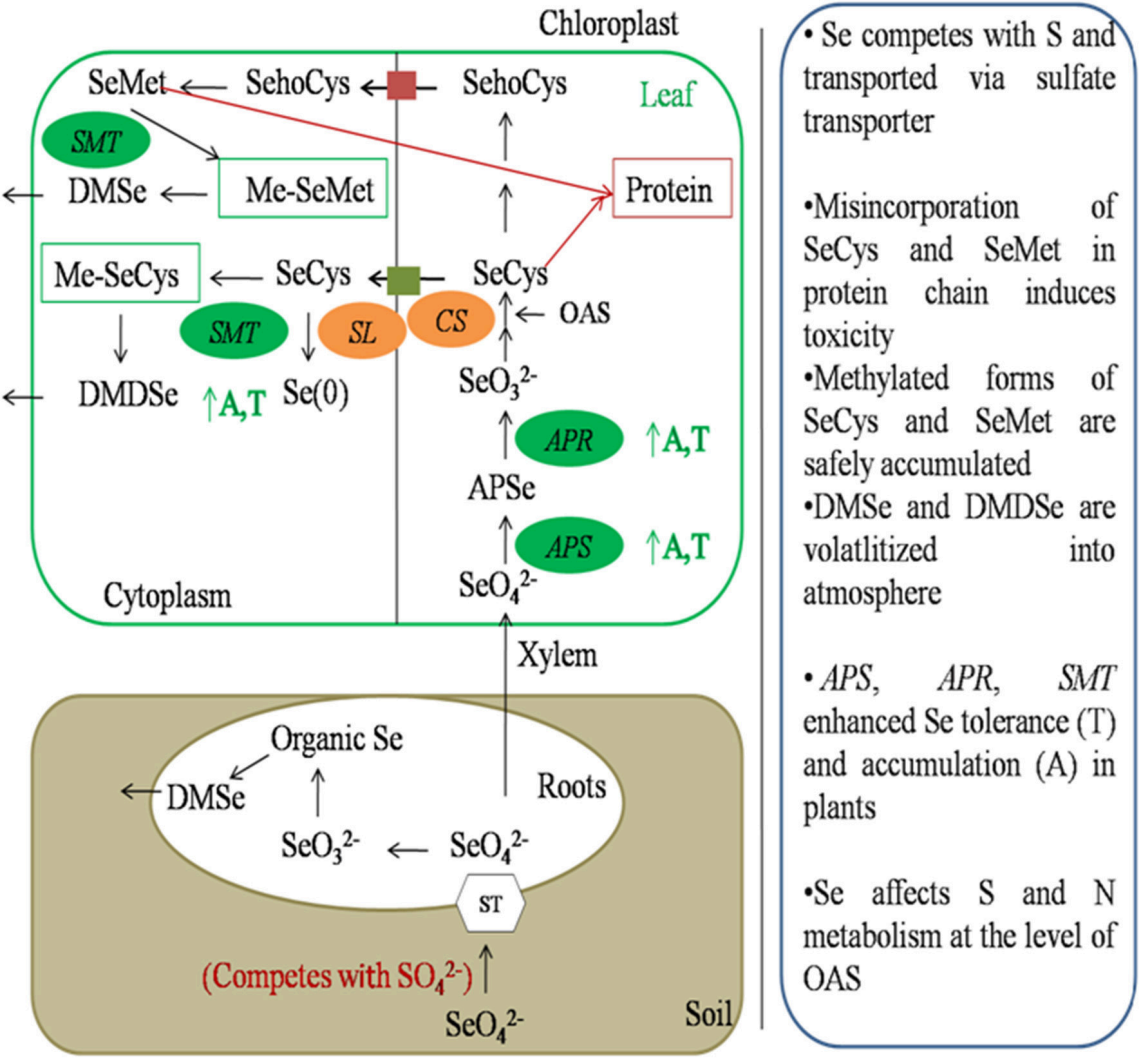
**Schematic representation of Se metabolism inside plant cells**. Selenate is transported inside the plant through sulfate transporters present in plasma membrane of roots. It is then transported to leaf through xylem. Selenate is then assimilated to DMSe and DMDSe by a series of sulfur metabolic enzymes. *APS*, ATP sulfurylase; *APR*, APS reductase; *CS*, Cysteine synthase; *SL*, Selenocysteine lyase; *SMT*, Selenocysteine methyl transferase; A, Accumulation; T, tolerance.

As Se is chemically similar to S, it competes with S and is transported inside the plant through sulfate transporters present in root plasma membrane (Sors et al., 2005; Li et al., 2008). After entry into plant, it is translocated to leaves and metabolized in plastids via sulfur assimilation pathway to SeCys or s SeMet. Sulfur analog of Se can be further methylated and vaporized into atmosphere in a non toxic form Pilon-Smits and Quinn (2010) (Figure 4).

First step in Se assimilation is conversion of inorganic Se to selenite. It requires the sequential action of two enzymes known as ATP sulfurylase (*APS*) and APS reductase (*APR*). *APS* catalyzes the hydrolysis of ATP to form adenosine phosphoselenate, which is further, reduced to selenite by *APR* (Sors et al., 2005). Selenite is then converted to selenide by enzyme sulfite reductase. In plants, this step can also be reduced by glutathione or glutaredoxins (Wallenberg et al., 2010). Selenide is then converted to SeCys by coupling with O-Acetyl serine (OAS) in the presence of an enzyme cysteine synthase. Cysteine-synthase has greater affinity for selenide as compared to sulfide. Depending upon plant species and environmental conditions SeCys can then be converted to elemental Se in the presence of enzyme SeCys lyase or can be methylated to methyl-SeCys (Me-SeCys) by selenocysteine methyltransferase or can be converted to selenomethionine (SeMet) by a series of enzymes.

Misincorporation of SeCys or SeMet in proteins leads to disruption of structure and function of protein, and cause Se toxicity in plants (Pilon-Smits and Quinn, 2010). SeMet can be used to form selenoproteins or methylated to form methyl-SeMet (Me-SeMet). Me-SeCys or Me-SeMet can be further volatilized into atmosphere as non-toxic dimethylselenide (DMSe) in non-hyperaccumulators or dimethyldiselenide (DMDSe) in hyperaccumulators (Pilon-Smits and Quinn, 2010; Figure 4)

## Selenium speciation in plants

*Brassica juncea* is a secondary Se accumulator which shows varied pattern of Se accumulation depending upon the type of Se specie absorbed. Uptake kinetics proved that selenate is more efficient than any other Se species. In SeMet enriched *Brassica* plants, Se-MeSeCys (Selenomethylselenocysteine) is the predominant Se accumulating specie followed by Se-Homocysteine and Se-Cystathionine. In selenate enriched *Brassica* plants, ion-pairing LC-ICP-MS was used to detect Se-speciation in which shoot extracts mostly consisted selenate, Se-MeSeCys and SeMet whereas, root extracts consisted selenate, selenite and SeMet. In selenite enriched plants, shoot extracts consisted of SeMetSeOxide hydrates as the predominant organic metabolite followed by selenite and SeMet whereas, root extracts showed the presence of SeMet and Se-MeSeCys. GC-MS technique confirmed the presence of volatilized DMSe and DMDSe in *Brassica* seedlings (Dumont et al., 2006).

In *Oryza sativa* accumulation of Se is mostly found to be in organic form i.e., SeMet followed by Se-MeSeCys and SeCys (Carey et al., 2012). Studies showed that in rice grain, Se is mostly concentrated in bran and is almost twice the levels of Se found in polished grain. The content of Se in rice decreased in the following trend: straw> bran> wholegrain > polished rice >husk (Sun H.-W. et al., 2010). In garlic, the most predominant form of Se is Se-MeSeCys, which accounts for most of the anticarcinogenic properties of garlic, followed by SeMet and SeCys. In onions also, Se-MeSeCys is the major form of Se speciation (Zhu et al., 2009). In Broccoli, Se-MeSeCys, selenate, selenite are the major forms of Se (Wu et al., 2015), whereas in mushrooms SeMet is the most accumulated form of Se (Dumont et al., 2006). In *Astragalus bisulcatus* plants, Se-MeSeCys is the main Se compound, whereas in seeds γ–glu–Se-MeSeCys (γ–glutamyl selnomethylselenocysteine) is the most predominant form. In Brazil nuts, SeMet is the most occurring Se compound (Dumont et al., 2006; Zhu et al., 2009). In grains such as wheat, rye, and barley SeMet is the most dominant Se compound (Stadlober et al., 2001; Poblaciones et al., 2014). Selenium hyperaccumulator *Stanleya pinnata* accumulated up to 90% of the total Se as Me-SeCys in plant tissues (Freeman et al., 2006).

## Beneficial effects of selenium in plants

**Figure 5:**
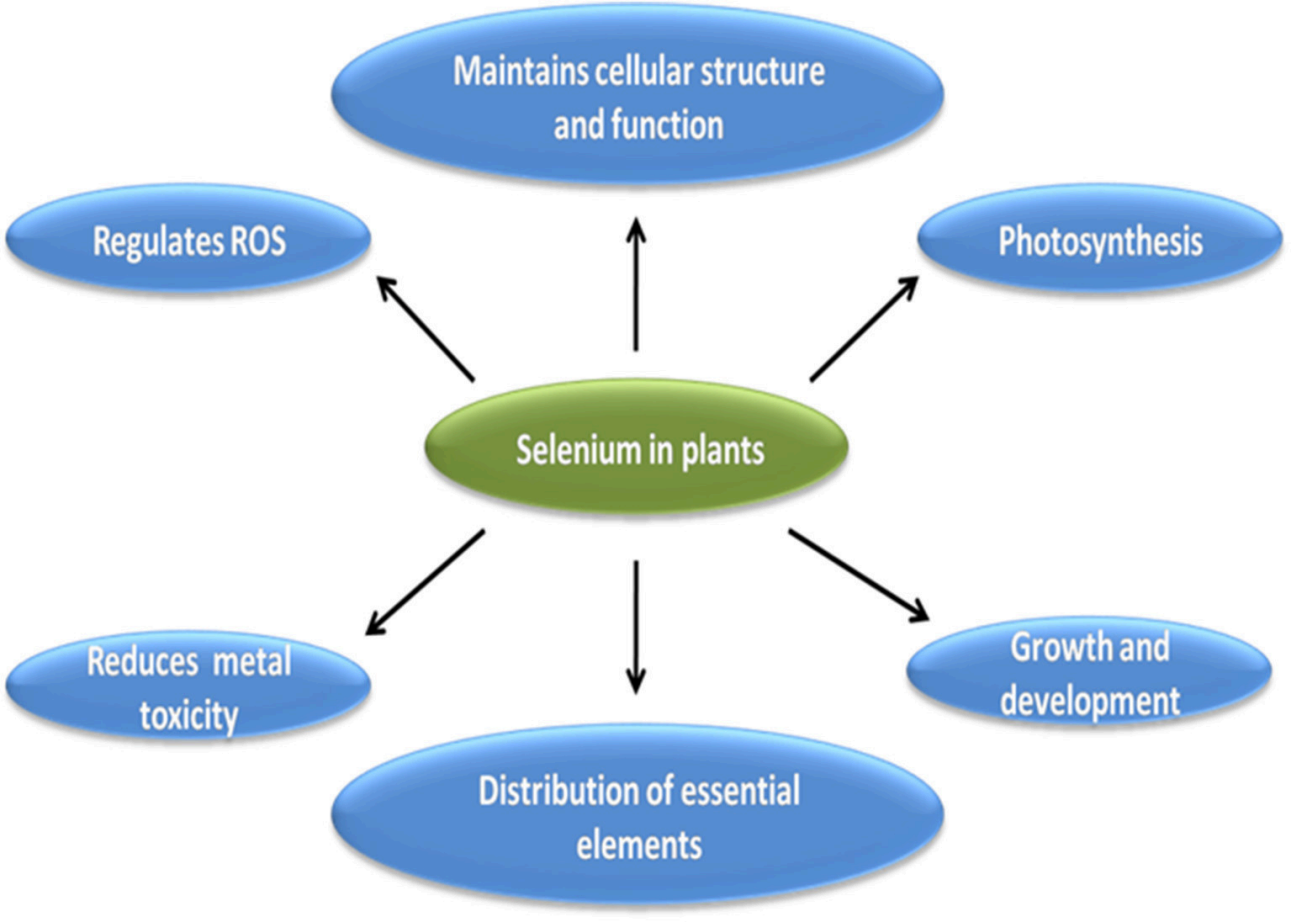
**Brief outline of beneficial effects of Se in plants**.

Although essentiality of Se to plants is in dilemma however, many workers have reported beneficial effect of Se in different plants (Cartes et al., 2010; Hasanuzzaman and Fujita, 2011; Pandey and Gupta, 2015). Brief outline of various role of Se in plants is described in pictorial form in Figure 5. All of the below mentioned roles of Se are interrelated to each other and contribute to overall growth and development of plant under stress and non-stressed conditions.

Studies have shown that Se at low doses protect the plants from variety of abiotic stresses such as cold (Chu et al., 2010), drought (Hasanuzzaman and Fujita, 2011), desiccation (Pukacka et al., 2011), and metal stress (Kumar et al., 2012; Pandey and Gupta, 2015). Under stress conditions, reactive oxygen species are produced in plants which disrupt cell membranes, proteins etc. Cartes et al. (2010) reported reduction in Al toxicity in rye grass by Se mediated dismutation of superoxide radical to H_2_O_2_. Kumar et al. (2012) reported reduction in ROS accumulation in Cd-stressed marine algae after application of 50 μM Se and Cd stressed *Brassica* seedlings after application of 2 μM Se (Filek et al., 2008). Similarly, decreased ROS accumulation was also reported in heat stressed *Sorghum* (Djanaguiraman et al., 2010), and As-stressed mungbean (Malik et al., 2012). However, at high doses, Se acts as pro-oxidant and causes oxidative stress in plants, for e.g., in Pb stressed roots of *Vicia faba* 1.5 μM Se decreased ROS but 6 μM increased ROS accumulation and decreased cell viability (Mroczek-Zdyrska and Wójcik, 2012). Filek et al. (2010) reported positive effect of 2 μM Se in maintaining the structure and fluidity of chloroplast membrane in Cd-stressed rape seedlings. Similar effect of low dose of Se on plastid membranes was also reported in Cd-stressed wheat seedlings (Filek et al., 2009), which could be attributed to reactivation of membrane enzymes or transportation of metabolites in chloroplast upon Se application. Se-mediated reduction in electrolytic leakage and improved cell integrity was also observed by many workers under various stress conditions (Zembala et al., 2010; Pukacka et al., 2011; Malik et al., 2012). Djanaguiraman et al. (2010) reported positive effect of low dose of Se on photosynthesis in *Sorghum*, which could be due to decreased ROS levels and increased antioxidant activity upon Se application. Similarly, Wang et al. (2012) found increased photosynthesis in rice seedlings at low doses but reported disrupted photosynthetic apparatus and photosynthesis at high dose of Se. Studies have reported beneficial role of Se in protecting the plants from heavy metal toxicity, which could be attributed to Se mediated detoxification of heavy metals due to less uptake, translocation or formation of non toxic Se-metal complexes (Belzile et al., 2006; Filek et al., 2008; Pedrero et al., 2008; Sun G.-X. et al., 2010; Zembala et al., 2010; Feng et al., 2011). Contrary to inhibited heavy metal uptake, active role of Se has been reported in Fe uptake (Feng R. W. et al., 2009; Feng and Wei, 2012), and could be considered as one of the Se mediated mechanism to reduce metal toxicity in plants, as Fe is an important constituent of chloroplast and photosynthetic electron transport chain (He et al., 2004). Apart from above mentioned roles, Se has been reported to delay senescence (Xue et al., 2001), increased yield in *Cucerbita pepo* (Germ et al., 2005), increased nutritive value of potato (Turakainen et al., 2006), increased respiratory potential in *Pisum sativum* (Smrkolj et al., 2006), chicory (Germ et al., 2007a) and *Eruca sativa* (Germ and Osvald, 2005), protecting the plants from pathogens, insects and herbivores (Freeman et al., 2006; Quinn et al., 2010).

## Selenium toxicity in plants

Selenosis or Se toxicity occurs in plants when optimum concentration of Se exceeds. Selenium causes toxicity by two mechanisms, one of which is malformed selenoproteins and another by inducing oxidative stress. Both the mechanisms are known to be harmful for plants in one or other way.

### Toxicity due to malformed selenoproteins

Malformed selenoproteins are formed due to the misincorporation of SeCys/SeMet in place of Cys/Met in protein chain. As compared to SeMet, substitution of SeCys is more reactive and detrimental to protein functioning. In a protein chain, cysteine residues play an important role in protein structure and function, and helps in formation of disulfide bridges, enzyme catalysis, and metal binding site. Hence, replacement of cysteine with SeCys is detrimental to protein structure and function as SeCys is larger, reactive and more easily deprotonated than cysteine (Hondal et al., 2012), as seen in case of methionine sulfoxide reductase function that got impaired after substitution with SeCys (Châtelain et al., 2013). SeCys substitution distorts tertiary structure of protein due to larger diselenide bridge formation and altered redox potential affect enzyme kinetics (Hondal et al., 2012). Fe-S cluster proteins of chloroplast and mitochondrial electron transport chain (Balk and Pilon, 2011) are prone to SeCys substitution for example as in case of chloroplast NifS-like protein (Pilon-Smits et al., 2002). Fe-Se cluster are larger in size and does not fit properly in apoproteins. Nitrogenase activity of *Klebsiella pneumonia* decreased five-fold after replacement of Fe-S cluster with Fe-Se (Hallenbeck et al., 2009). However, in another study substitution reaction proved to be beneficial for glutathione dependent peroxidase in *Citrus sinensis* (Hazebrouck et al., 2000). Several studies have shown that diversion of selenocysteine from protein synthesis is associated with enhanced Se tolerance in plants for e.g., overexpression of SeCys-methyltransferase in *Arabidopsis* and *Brassica juncea* (LeDuc et al., 2004), Cystathionine gamma-synthase, NifS-like protein with selenocysteine lyase activity in *Brassica juncea* (Van Huysen et al., 2003; Van Hoewyk et al., 2005).

### Se toxicity due to oxidative stress

At high doses, Se acts as pro-oxidant and generates reactive oxygen species which cause oxidative stress in plants. Generally, under Se stress decreased level of glutathione is observed (Hugouvieux et al., 2009), except in Se-tolerant plants where elevated levels are observed (Grant et al., 2011). In another study by Grant et al. (2011) on *cad2-1* mutant plants having defective glutathione synthetic pathway showed many folds decreased root length as compared to wild type plants grown on 20 μM selenate. In apr2-1 mutant of *Arabidopsis*, glutathione depletion and ROS accumulation found to be interlinked to each other under Se stress (Grant et al., 2011). Increased lipid peroxidation was observed in wheat seedlings under Se stress (Łabanowska et al., 2012). Several studies have reported increased activity of antioxidant enzymes indicating ROS accumulation under Se stress (Gomes-Junior et al., 2007; Chen et al., 2008; Akbulut and Cakır, 2010; Schiavon et al., 2012). Tamaoki et al. (2008) found higher accumulation of ROS in *vtc1* mutant having defective ascorbic acid biosynthetic pathway as compared to wild type plants under Se stress. In another study, Se generated ROS initiated defense mechanism against Se stress (De Pinto et al., 2012). Previous studies reported that ROS accumulation under Se stress increased lipid peroxidation, cell mortality in *Arabidopsis* and *Vicia faba* (Lehotai et al., 2012; Mroczek-Zdyrska and Wójcik, 2012). Apart from plant cells, Wallenberg et al. (2010) reported generation of mitochondrial superoxide in human cells under Se stress. Altogether, above studies indicate role of reactive oxygen species in imparting Se toxicity in plants.

## Selenium phytoremediation

Phytoremediation is a plant based technology, in which toxic metals are removed from the soil by the roots of the plant. Further, metals translocated to the upper parts of the plant from where they can be easily removed by harvesting, or volatilized into atmosphere in less toxic forms (Newman and Reynolds, 2004). This method of cleaning up of soil is cheaper, and it does not reduce the fertility of the soil like other engineering methods (Robinson et al., 2000; Pilon-Smits and Freeman, 2006). Due to natural and anthropogenic activities Se pollution is increasing in certain regions of the world (Hamilton, 2004; Hira et al., 2004). Studies have shown increasing use of plants to counteract Se pollution in the environment. Plants volatilize the accumulated Se as DMSe and DMDSe, which are almost 600 times less toxic than elemental Se (Dumont et al., 2006). Apart from terrestrial plants (Kahakachchi et al., 2004), macrophytes such as muskgrass, *Phragmites australis* (Shardendu et al., 2003), and *Potamogeton crispus* (Wu and Guo, 2002) had been used to clean Se present in agricultural drainage water (Lin et al., 2002). *Brassica* species have been known to accumulate and volatilize Se (Bañuelos et al., 2005, 2007). *Stanleya pinnata* and *Astragalus bisulcatus* are well-known Se-accumulators; however, slow growth rate and low biomass production often limit their phytoremediation potential (Germ et al., 2007b). In hyperaccumulators, Se is detoxified by methylation of SeCys and SeMet to Me-SeCys and Me-SeMet which are non-toxic and accumulated safely. Methylation occurs in presence of enzyme selenocysteine methyltransferase (Pilon-Smits and LeDuc, 2009).

**Figure 6:**
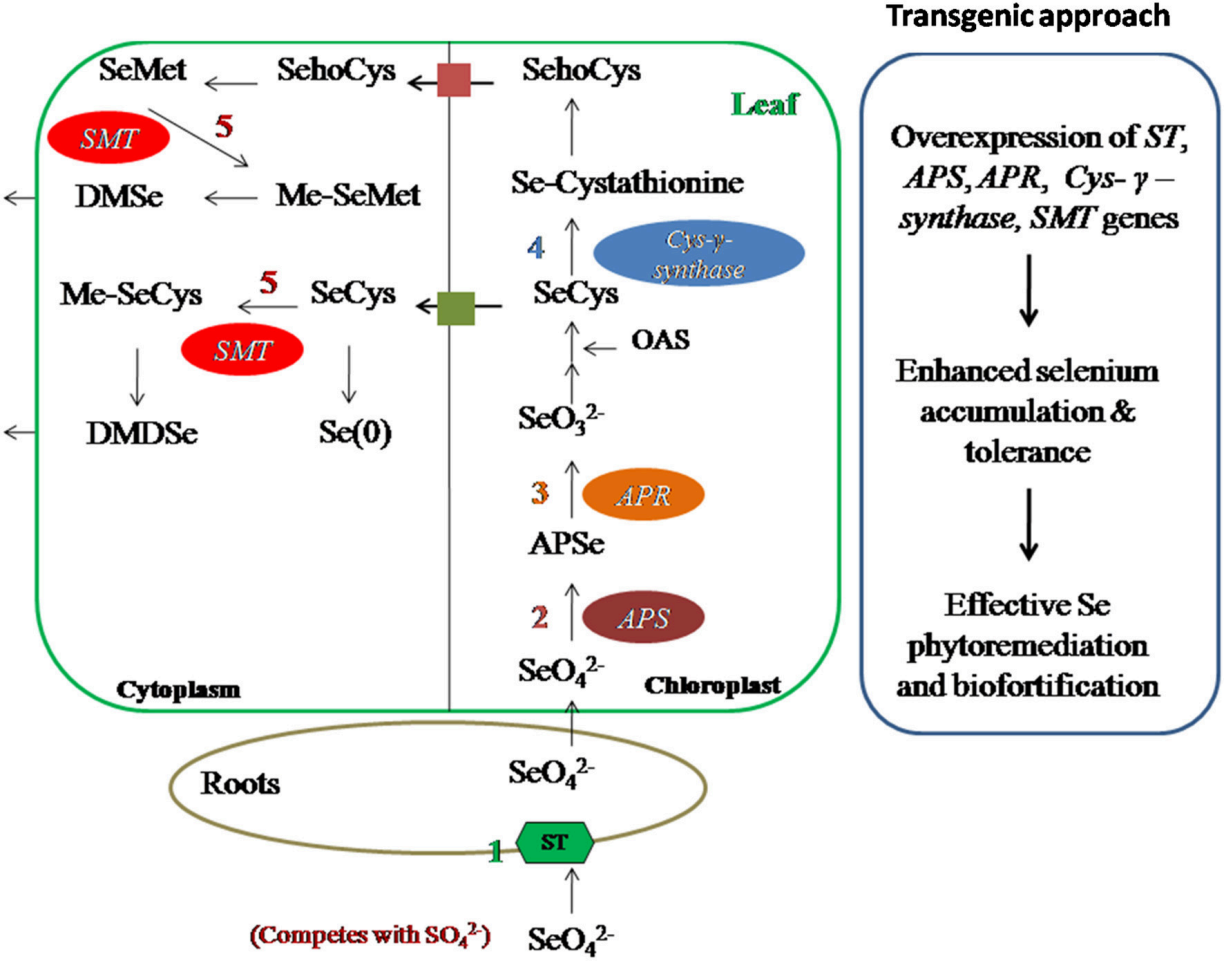
**Transgenic approach to improve Se-phytoremediation and biofortification**. Overexpression of ST, APS, APR, Cys–γ–synthase, SMT genes have shown enhanced Se accumulation and tolerance in different plants hence this technique can be used for Se phytoremediation and biofortification in Se-toxic and Se-deficient regions, respectively. ST, sulfur transporter; APS, ATP sulfurylase; APR, APS reductase; Cys–γ–synthase, Cystathionine–γ–synthase; SMT, Selenocysteine methyl transferase.

Phytoremediation efficiency of plants has been enhanced using genetic engineering (Eapen and D'souza, 2005; Meagher and Heaton, 2005; Figure 6). Overexpression of ATP sulfurylase (*APS1*) of *Arabidopsis thaliana* in *Brassica juncea* increased selenate reduction along with two- to three- fold increase in Se accumulation in shoots and roots (Pilon-Smits and LeDuc, 2009). Overexpression of mouse selenocysteine lyase in *Arabidopsis thaliana* and *B. juncea* resulted in higher Se accumulation and tolerance as compared to wild type plants (Garifullina et al., 2003). Overexpression of cystathionine-γ-synthase of *Arabidopsis* enhanced two- to three- folds Se volatilization in *B. juncea* (Van Huysen et al., 2003). Double transgenic plants obtained by expressing both APS and SMT gene showed 9 times higher Se accumulation than wild type plants. All transgenic plants showed promising traits of enhanced Se accumulation, volatilization, and tolerance which are needed for effective and efficient phytoremediation of Se.

## Selenium biofortification

Although phytoremediation is an efficient method of cleaning up of soil from Se, but the problem still exists regarding the disposal of the contaminated plant material which could be toxic to human and animal survival if left as such. Biofortification is an alternative method to dispose-off these waste plant material (Liu et al., 2011; Lin et al., 2014). In this strategy, Se enriched plant material will be decomposed in agricultural soil which can be used further for the enrichment of food products with Se (Bañuelos et al., 2015). Hence, biofortification is a practice of enriching the agricultural food products with certain nutrients, for example Se, to increase the dietary intake through plant breeding, genetic engineering and manipulation of agronomic practices (Zhu et al., 2009; Kieliszek and Blazejak, 2012; Borrill et al., 2014). Biofortification is an economical safe agricultural technique, which aims to cope up with deficiency of a particular nutrient in diet, and increase the content of a micronutrient for e.g., Se in edible portion of plant (Nestel et al., 2006; Mayer et al., 2008; Zhao and McGrath, 2009). Selenium biofortification substantially increases Se contents of agricultural food products, and can help in alleviation of Se malnutrition to which more than 1 billion people all over the world is suffering (WHO, 2009). Researchers round the world are trying to develop Se-enriched food products to minimize Se related deficiency disorders. Selenium fertilization also affects the synthesis of amino acids, protein and phenolics compounds. Selenium-biofortified tomato fruit has been reported to have high contents of flavonoids and leaf phenolic contents (Schiavon et al., 2013). Selenium biofortification affects the synthesis of glucosinolates (GLS), S-secondary compounds, which on hydrolysis produce isothiocyanates having anticancerous properties (Dinkova-Kostova, 2013). *Brassica* species are rich source of GLS (Robbins et al., 2005; Barickman et al., 2013), however, high levels GLS may be toxic to humans and animals (Tripathi and Mishra, 2007). As different plants have different Se accumulation capacity (Galeas et al., 2007), hence, to produce Se-biofortified food products it is very important to select those plant species that can moderately accumulate Se in their edible parts for e.g., Se levels in different plants are of following order: *brassica* > bean > cereal (Liu et al., 2011). The rice cultivars Jinlong No.1 and Longquin No.4, accumulate more Se than ordinary normal rice, and are naturally Se enriched. These cultivars are being cultivated on large scale in Se deficient regions of China (Yang et al., 2007; Yin and Yuan, 2012; Wang J. W. et al., 2013).

Foliar application of Se is better and efficient means of Se-biofortification than application of Se fertilizers in soil, due to avoidance of root to shoot translocation of Se (Winkel et al., 2015). Use of Se fertilizers in soil have low rates of Se enrichment in edible part of plant, moreover, long term use can be toxic to nearby ecosystem, hence use of Se fertilizers should be done carefully to avoid toxic aspects (Winkel et al., 2015). In Finland and New Zealand, use of inorganic Se fertilizers is a common practice to increase Se content in agricultural products (Schiavon et al., 2013; Wang Y. D. et al., 2013). Implementation efficiency of Se fertilizers for Se-biofortification strategy can be increased by the use of organic acids (Morgan et al., 2005; Lynch, 2007), organic forms of Se (Schiavon et al., 2013; Pezzarossa et al., 2014), or microbes (Duran et al., 2013, 2014) which enhance the chances of Se availability to plants. Genetic engineering is another useful strategy to obtain Se-biofortified food products, which generally focuses on manipulation of Se-related enzymes for Se uptake, assimilation and volatilization (Figure 6, Box 2). *Brassica juncea* plants over-expressed with *Astragalus bisulcatus SMT* gene (selenocysteine methyltransferase) and *Arabidopsis thaliana* ATP sulfurylase gene showed significantly improved Se accumulation and tolerance than wild type plants (LeDuc et al., 2004, 2006).

Box 2 **Transgenic approach.**

Selenium metabolism inside the plants can be manipulated using transgenic approach for effective Se-phytoremediation or biofortification. Prime focus should be to:

- Increase plant tolerance of high soil Se concentration.
- Increase Se uptake and transport to shoot.
- Increase Se accumulation in shoot tissues.
- Increase Se volatilization.

Wheat is the most efficient accumulator of Se within the common cereal crops (wheat>rice>maize>barley>oats) and in cereals SeMet is the dominant organic form of Se (Stadlober et al., 2001; Poblaciones et al., 2014). Wheat is also one of the most important sources of dietary Se for human population in UK (Lyons, 2010). Thus, wheat is an obvious target crop for agronomic biofortification to increase the dietary Se intake, and thus the Se status of UK population. Studies conducted by Cubadda et al. (2010) showed that wheat collected from seleniferous belt of Nawanshahr-Hoshiarpur region in Punjab (India), have high concentrations of Se ranging from 29 to 185 μg/g. The regular consumption of such wheat can produce Se toxicity, but can be used as Se supplement in diet in low Se areas.

Rice, being the staple food crop for more than half of world's population, is an important source of Se especially for inhabitants of China who depend on it for their nutritional requirements (Chen et al., 2002; Hu et al., 2002). China stands high as Se rich country (ranked fourth) however, Se deficiency is also observed in certain regions, for instance Heilongjiang and Yunnan Province where Se-fortified wheat, rice, and vegetables are the primary source of Se in diet (Zhu et al., 2009; Liu et al., 2011). Significant increase in Se content of rice grains have been reported by foliar applications of Se-enriched fertilizers (Pezzarossa et al., 2012; Boldrin et al., 2013). High content of Se ranging between 15 and 270 μg/kg DW was observed in legumes in Spain (Matos-Reyes et al., 2010). Peas are the best candidate among legumes to carry out Se biofortification under Mediterranean conditions (Poblaciones et al., 2012).

## Future prospects

In this review, we have addressed the basic mechanism of uptake, metabolism, and toxicity of Se in plants including phytoremediation and biofortification aspects. But still there are many faces of Se which needs to be uncovered. The beneficial effect of Se at low doses also mentioned, however, the exact mechanism behind the effect is still untouched. We also need to explore in detail how S and Se biochemistry are interlinked, and influence each other during Se uptake. Focus on enzyme kinetics of various steps of S assimilatory pathway at different concentrations of Se, also need to be explored. In addition, how different plants have different Se tolerance and detoxification mechanisms, and exploitation of these mechanisms to improve phytoremediation and biofortification of Se, also needs to be uncovered by integrating both the approaches. Furthermore, product of phytoremediation can become raw material for biofortification purposes, hence, it is very important to screen Se-hyperaccumulator plants and those plant species that can accumulate Se in edible parts within the safer limits for human consumption.

## Author contributions

MG planned, drafted and checked the manuscript. SG designed, wrote and executed the manuscript.

### Conflict of interest statement

The authors declare that the research was conducted in the absence of any commercial or financial relationships that could be construed as a potential conflict of interest. The reviewer SM and handling Editor declared their shared affiliation, and the handling Editor states that the process nevertheless met the standards of a fair and objective review.
